# Implications of Climate Change: How Does Increased Water Temperature Influence Biofilm and Water Quality of Chlorinated Drinking Water Distribution Systems?

**DOI:** 10.3389/fmicb.2021.658927

**Published:** 2021-06-08

**Authors:** Carolina Calero Preciado, Joby Boxall, Víctor Soria-Carrasco, Soledad Martínez, Isabel Douterelo

**Affiliations:** ^1^Department of Civil and Structural Engineering, Sheffield Water Centre, The University of Sheffield, Sheffield, United Kingdom; ^2^NERC Biomolecular Analysis Facility, Department of Animal and Plant Sciences, The University of Sheffield, Sheffield, United Kingdom; ^3^Department of Animal and Plant Sciences, The University of Sheffield, Sheffield, United Kingdom; ^4^Área de Microbiología, Departamento de Biociencias, Facultad de Química, Universidad de la República, Montevideo, Uruguay

**Keywords:** biofilms, discolouration, bacteria-fungi, opportunistic pathogens, temperature

## Abstract

Temperature variation can promote physico-chemical and microbial changes in the water transported through distribution systems and influence the dynamics of biofilms attached to pipes, thus contributing to the release of pathogens into the bulk drinking water. An experimental real-scale chlorinated DWDS was used to study the effect of increasing temperature from 16 to 24°C on specific pathogens, bacterial-fungal communities (biofilm and water samples) and determine the risk of material accumulation and mobilisation from the pipes into the bulk water. Biofilm was developed for 30 days at both temperatures in the pipe walls, and after this growth phase, a flushing was performed applying 4 gradual steps by increasing the shear stress. The fungal-bacterial community characterised by Illumina MiSeq sequencing, and specific pathogens were studied using qPCR: *Mycobacterium* spp., *Mycobacterium avium* complex, *Acanthamoeba* spp., *Pseudomonas aeruginosa*, *Legionella pneumophilia*, and *Stenotrophomonas maltophilia*. Sequencing data showed that temperature variation significantly modified the structure of biofilm microbial communities from the early stages of biofilm development. Regarding bacteria, *Pseudomonas* increased its relative abundance in biofilms developed at 24°C, while fungal communities showed loss of diversity and richness, and the increase in dominance of *Fusarium* genus. After the mobilisation phase, *Pseudomonas* continued being the most abundant genus at 24°C, followed by *Sphingobium* and *Sphingomonas.* For biofilm fungal communities after the mobilisation phase, Helotiales *incertae sedis* and *Fusarium* were the most abundant taxa. Results from qPCR showed a higher relative abundance of *Mycobacterium* spp. on day 30 and *M. avium* complex throughout the growth phase within the biofilms at higher temperatures. The temperature impacts were not only microbial, with physical mobilisation showing higher discolouration response and metals release due to the increased temperature. While material accumulation was accelerated by temperature, it was not preferentially to either stronger or weaker biofilm layers, as turbidity results during the flushing steps showed. This research yields new understanding on microbial challenges that chlorinated DWDS will undergo as global temperature rises, this information is needed in order to protect drinking water quality and safety while travelling through distribution systems.

## Introduction

Drinking water distribution systems (DWDS) are not sterile environments (Liu et al., 2013) and it is well known that most of the microorganisms inhabiting these systems live attached to internal pipe surfaces forming biofilms (Batté et al., 2003; Douterelo et al., 2014a). The biofilm mode of life confers a series of advantages to its members such as protection against disinfectants, facilitates exchange of genetic material or enhancing metabolic capacities helping the acquisition of nutrients (Flemming, 2002). The mobilisation of microorganisms from biofilms into the bulk water has been associated with several problems in DWDS, including discolouration, taste or odour, increase of metals, release and proliferation of potential pathogens (Szewzyk et al., 2000; Douterelo et al., 2014a; Nescerecka et al., 2014; Husband et al., 2016). Biofilm development in DWDS is affected by several factors, such a pipe material, hydraulic conditions, the amount and type of disinfectant or the concentration of organic and inorganic compounds (Batté et al., 2003; Douterelo et al., 2013; Mi et al., 2015; Ren et al., 2015). Another key factor affecting DWDS is temperature, which is an key parameter of water quality and can modify processes occurring within DWDS (Delpla et al., 2009; Bondank et al., 2018). Temperature is an important determinant of water quality, since it influences physical, chemical and biological processes, such as absorption of chemicals, chlorine decay (Hua et al., 1999) and microbial growth and competition processes (Nescerecka et al., 2014). Currently, worldwide we are experiencing more frequent extreme weather events, with high temperature peaks during short periods of time, especially in regions with desert and continental-like climates such a Mediterranean countries (Mesquita et al., 2013; World Health Organization (WHO), 2017). As a consequence of climate change, these temperature peaks could be of greater intensity, longer, and they could happen more often in other climatic zones (World Health Organization (WHO), 2017).

However, limited knowledge exists on how temperature affects biofilms in chlorinated DWDS when decay of chlorine due to higher temperatures can facilitate further biofilm growth (Hua et al., 1999; Li et al., 2018) and select for certain microorganisms including pathogens. Changes in temperature in DWDS are strongly influenced by weather, soil type, depth of installation of pipes and hydraulic residence times (Agudelo-Vera et al., 2020). Zlatanović et al. (2017), while studying domestic non-chlorinated Dutch copper pipe systems, showed the influence of temperature on bacterial communities under stagnation. Similarly, a recent study also in an unchlorinated system has shown that temperature rise associated to the use of cold recovery technology lead to biomass and bacterial community changes in these systems (Ahmad et al., 2020). However, these studies in non-chlorinated systems focus only on bacteria, they do not represent the dynamics of typical chlorinated DWDS, and they do not contribute either to understand the risks associated to biofilm mobilisation. Furthermore, several studies have demonstrated the incidence of opportunistic pathogens (OPs) such a non-tuberculous mycobacteria (NTM) or *Legionella* spp. in DWDS, and its ability to proliferate in biofilm in drinking water systems (van der Wielen and van der Kooij, 2013; Ashbolt, 2015). In different freshwater ecosystems such a rivers, lakes, reservoirs or groundwater it has been observed that the growth of some of these pathogens depends on the temperature (Pandey et al., 2014). In unchlorinated DWDS, it has been suggested that higher temperature in the summer could have contributed to the growth of OPs such a Mycobacteria or *L. pneumophila* (van der Wielen and van der Kooij, 2013). The present research provides new understanding in relation to the impact of temperature on biofilms and the potential mobilisation of microorganisms and in particular pathogens (including amoeba and fungi) in a chlorinated DWDS under representative hydraulic regimes.

## Materials and Methods

### Experimental DWDS Facility and Experiment Conditions

In order to ensure controlled yet fully representative conditions, the research was carried out using a temperature controlled full scale experimental DWDS facility at the University of Sheffield (United Kingdom), previously described in detail by Douterelo et al. (2014b) (Supplementary Figure 1A). In brief, the facility consists of 3 independent recirculating loops of High-Density Polyethylene (HDPE). Each pipe loop consists of 9.5 m × 21.4 m long coils of 79.3 mm internal diameter HDPE pipe, thus has a total length of 203 m. HDPE was selected as it is a prevalent material used in DWDS world-wide (World Health Organization (WHO), 2006). The system allowed for experimental replication by running three independent loops simultaneously. The water running through the system came from the local drinking water supply (an upland source with a ferric based treatment process and then 36″ diameter cast iron mains before entering the laboratory via a dedicated pipe) thus representing realistic conditions. Water entering the system was heated via two heaters wired in series installed in each tank (Redring P27DC, United Kingdom), warming the water at the temperature needed for the experiments. The facility has removable coupons that can be inserted into the pipes to enable *in situ* analysis of biofilms (Deines et al., 2010). Each coupon has an outer part for molecular analysis and an insert for microscopy, making possible simultaneous visual and molecular analysis of biofilms without modifying or destroying the biofilm (Supplementary Figure 1B; Deines et al., 2010). Before the experiments started, the whole facility was disinfected with 20 mg/L of RODOLITE H (RODOL Ltd., United Kingdom), a sodium hypochlorite based solution with less than 16% free chlorine, following the protocol described by Douterelo et al. (2014b). After disinfecting the system, sterile coupons were inserted along the pipes.

To study the effect of temperature fluctuation on the DWDS microbiome (biofilm and planktonic communities) and the potential of biofilm mobilisation from the pipe into the bulk water, experiments were performed first at 16 and at 24°C, respectively. Please note that the 3 independent loops were run to obtain replication for each experiment. This temperature selection was within the realms of the climate change predictions and based on previous research showing that the average water temperature in UK DWDS is 16°C in spring-summer months (Husband et al., 2008) and on the WHO recommendation that the temperature of drinking water should not exceed 25°C to limit microbial growth (World Health Organization (WHO), 2017).

Each experiment comprised of a biofilm growth phase of 30 days, followed by a flushing programme used to study the mobilisation dynamics of biofilms from pipe surfaces (Supplementary Table 1 and Supplementary Figure 2). The biofilm growth phase of 30 days was selected based on previous studies in the same facility that showed this time as sufficient to detect and monitor discolouration and biofilm mobilisation events, as well as microbial community changes (Douterelo et al., 2013, 2014b; Fish and Boxall, 2018; Rosales et al., 2020).

During the biofilm growth phase, the facility was operated using a representative low varied flow hydraulic regime (ranging from 0.2 to 0.5 L/s) based on daily patterns observed in real DWDS in the United Kingdom (Husband et al., 2008). Similarly, the residence time of the water in the system was set to 24 h, thus representing conditions in actual distribution systems in the United Kingdom. After the 30 days of biofilm growth phase, the system was flushed, using the same protocol to flush the pipes for both temperature conditions. The flushing protocol consisted in 4 gradual steps by increasing the shear stress (τ) in the pipes to explore if the strength profile of the biofilms were altered by the temperature changes: step 1 = 0.4 N/m^2^, step 2 = 2.3 N/m^2^, step 3 = 3.4 N/m^2^, step 4 = 4.3 N/m^2^ (Supplementary Table 1 and Supplementary Figure 2). Each step was performed for a duration of 3 water turnovers (i.e., the time that the total volume of the water needs for recirculate in the loop three times), to mix and detect the maximum amount of material mobilised from the pipes into the bulk water (Sharpe et al., 2010).

### Microbial Communities Sampling and Water Quality Physico-Chemical Conditions During Biofilm Growth Phase and Mobilisation Experiments

To study temporal dynamics of planktonic and biofilm communities, water samples from each loop and coupons were obtained on day 0 when the experimental conditions were optimal, and the water had reached the study temperature. This took around 4 h for each temperature, from when the water filled the loop until stable temperature values were reached and representative samples of day 0 were taken. Then biofilm and bulk water samples were taken every 10 days until day 30 during the growth phase at both temperatures. This monitoring approach allowed pair-wise comparisons of microbial community succession under the two temperatures over time from early stages of biofilm development. Water and biofilm samples were also obtained after the mobilisation events at both temperatures. In each sampling event, 3 replicates of 2 L of bulk water were taken from sampling taps located in each loop and they were filtered through a 0.22 μm nitrocellulose membrane filters (Millipore-Corp., United States). For the biofilm samples, 9 coupons were taken in each sampling point to obtain 3 biological replicates by pooling biofilms from 3 coupons. Biofilms were removed from coupons following a standardised protocol as described in Fish et al. (2015), creating a biofilm suspension that was filtered through a 0.22 μm nitrocellulose membrane filters (Millipore-Corp., United States). Filters of water and biofilm samples were preserved in the dark and at −20°C prior to subsequent DNA extractions. In total, *n* = 30 biofilm samples and *n* = 30 water samples were analysed. No DNA was extracted from the coupon samples taken on day 0, indicating that there was no biological material developed in the coupon.

Turbidity, iron (Fe) and manganese (Mn) concentrations were studied as the main parameters associated with discolouration (Seth et al., 2004) and total organic carbon (TOC) to test the organics in water. Turbidity was measured online every minute during the experiments by an ATi-A15/76 turbidity monitor (ATi, United Kingdom), installed in the experimental facility. At the time of sampling, pH and water temperature were measured in triplicate using a Hanna portable metre HI-991003 (Hanna Instruments, United Kingdom). Free and total chlorine were tested with a Palintest-CS100 chlorosense (Palintest, United Kingdom). In addition, at each sampling event 3 replicates of discrete water samples were taken to analyse TOC, and the concentration of Fe and Mn at The Kroto Research Institute (KRI) (The University of Sheffield, United Kingdom). Samples for TOC were stored in 20 ml glass vials and then analysed using a Shimadzu TOC-V_CPH_/_CPN_ Analyzer (Shimadzu, Japan) following manufacturer’s instructions. For Fe and Mn concentrations, water samples were collected in 20 mL vials containing 5M of nitric acid and then ions were monitored by means of an Inductively Coupled Plasma Mass Spectrometry (ICP-MS) on a Perkin Elmer Elan DRC-II (PerkinElmer, United States) (Sloetjes and Wittenburg, 2008).

### Microscopy Analysis

Visual characterised of biofilms enable qualitative assessment of overall differences between temperatures. Coupon inserts from day 0 (control) and day 30 were analysed in triplicate by Scanning Electron Microscopy (SEM), at The Faculty of Science, Electron Microscopy Facility at the University of Sheffield (United Kingdom). Samples were fixed in 5% formaldehyde solution (Fisher Scientific, United Kingdom) for 24 h and preserved in Phosphate Buffer Solution (PBS) (Gibco^®^, Thermo Fisher Scientific, United Kingdom), at 4°C. Afterward, samples were fixed secondary following the protocol described by Fischer et al. (2012). Samples were coated with approximately 25 nm of gold in an Edwards Gold Sputter Coater S150B (Edwards, United Kingdom). Micrographs of biofilms were obtained with a TESCAN-Vega3 LMU (Girton, Cambridge, United Kingdom) at an accelerating voltage of 15 kV.

### DNA Extraction and Sequencing

DNA from all the filters with concentrates of water and biofilm samples was extracted following the protocol based on hexadecylmethylammonium bromide (CTAB) and proteinase K chemical lysis, followed by DNA purification using phenol/isoamyl alcohol method (Zhou et al., 1996; Neufeld et al., 2007). DNA concentration from each extraction was then quantified fluorometrically using the HS dsDNA Assay kit with a Qubit 4 Fluorometer (Invitrogen, United States). Extracted DNA was sequenced by Mr DNA Laboratory (Shallowater, TX, United States) on the Illumina MiSeq platform following the manufacturer’s guidelines for pair-end sequencing. Bacterial 16S rRNA gene fragments were amplified using the primers 28F (5′-GAGTTTGATCNTGGCTCAG-3′) and 519R (5′-GTNTTACNGCGGCKGCTG-3′) spanning the V1 to V3 hypervariable regions. For fungal analysis, primers ITS1FBt1 (5′-CTTGGTCATTTAGAGGAAGTAA-3′)/ITS2R (5′- GCTGCGTTCTTCATCGATGC-3′) targetting the ITS1-2 regions were selected for amplification. Sequencing data were deposited in the NCBI Sequence Read Archive (SRA) with the accession number PRJNA656259.

### Bioinformatics and Community Analysis

An initial quality control of the sequencing raw data was carried out using the FastQC software v0.11.8 (Andrew, 2010). BBDuk software v37.95 was used to remove sequencing errors (Davis et al., 2018) and to filter and trim sequences with an average quality phred score below 20 and/or a minimum length of 100 bp (Cock et al., 2009). Sequencing reads, with a phred score of at least 20 and a length between 100 and 300 bp, were demultiplexed and depleted of barcodes by applying the sabre software (Joshi, 2011) and imported into the Quantitative Insights Into Microbial Ecology 2 program v2019.7 (QIIME2) (Bolyen et al., 2019). Then, pair-end sequences were joined and dereplicated, chimeric sequences were identified and filtered and *de novo* clustering by 97% similarity was performed to obtain the Operational Taxonomic Units (OTUs) using the vsearch plug-ins in QIIME2. See Supplementary Tables 2, 3 for more information on the sequence count in each step of the bioinformatic analysis. The taxonomic assignment of the final OTUs was carried out using the classify-consensus-vsearch method (Rognes et al., 2016) of the feature-classifier plug-in in QIIME2 (Bokulich et al., 2018). 16S rRNA sequences were compared against the SILVA SSU r132 database (Quast et al., 2013) and ITS2 sequences against UNITE 8.0 (Kõljalg et al., 2013).

Rarefied tables based on of the relative abundance of 97% OTUs for both bacteria and fungi, were used to calculate alpha- and beta-diversity (Morris et al., 2014). Alpha-diversity, which measures the internal diversity of each sample, was calculated as a measurement of Chao 1 index (richness estimator), Simpson index (dominance), and Shannon index (diversity) (Morris et al., 2014) using the rarefied OTU tables at 97% cut-off with the q2-diversity plug-in in QIIME2. For beta-diversity, which estimates the degree of differentiation between samples, the rarefied OTU tables at 97% cut-off was square-root transformed and then the Bray-Curtis method was applied to construct similarity matrices using the vegan package v2.5-6 in R (Oksanen et al., 2019). Bray-Curtis resemblance matrices were visualised by non-metric multidimensional scaling (nMDS) plots with ggplot2 package v3.2.1 in R (Wickham and Chan, 2016).

### Quantitative PCR (q-PCR)

q-PCR was performed to monitor changes in the gene copy number of 6 OPs previously reported in DWDS (van der Wielen and van der Kooij, 2013; Qin et al., 2017; Liu et al., 2019). At genus level, the 16S rRNA gene of *Mycobacterium* spp. was targetted because many of their members of this NTM are responsible for causing a large number of diseases (Liu et al., 2016). The 18S rRNA gene of *Acanthamoeba* spp. was selected since this genus of free-living amoeba act as an important host of other pathogens in drinking water (Dobrowsky et al., 2016). At species level, the 16S rRNA gene of *Mycobacterium avium* complex, regA gene of *Pseudomonas aeruginosa*, mip gene of *Legionella pneumophilia*, and chiA gene of *Stenotrophomonas maltophilia* were quantified for having a global importance for human health and because their recognition as major agents of concern in drinking waters are currently increasing (Ashbolt, 2015; Benedict et al., 2017).

The amplification, detection and quantification were performed in a QuantStudio^TM^ 12K Flex Real-Time PCR System (Thermo Fisher Scientific Inc., United Kingdom), following the protocol described by van der Wielen and van der Kooij (2013). Absolute quantifications were based on comparison of the value of sample cycle threshold (CT; i.e., the number of PCR cycles needed for the fluorescence signal of the amplified DNA to cross the fluorescence threshold, which is significantly above the background fluorescence) with the CT value of a calibration curve, based on known copy numbers of the target microorganisms (Kubista et al., 2006; van der Wielen and van der Kooij, 2013; Biosystems, 2015).

### Statistical Analysis

To determine the normality of all data sets the Shapiro-Wilks test was applied (Fowler et al., 2013). Statistical differences between parameters (physico-chemical, alpha-diversity values, and gene number of OPs) were tested via the non-parametric test Mann-Whitney *U test*. For beta-diversity, the analysis of similarities (ANOSIM) test was applied to Bray-Curtis distance matrices based on OTUs relative abundance to detect statistically significant differences in biofilm and water microbial community structures between temperatures. The global-R statistic values obtained from this analysis showed the strength of the impact of temperature on bacterial and fungal community structure. Global-R values ranges from 0 to 1, where 1 indicates that communities are totally different (Anderson and Walsh, 2013; Douterelo et al., 2018). Differences were considered statistically significant when *p-value* was ≤0.05, and all statistical tests were carried out using R-v3.6.1 (R Core Team, 2014).

## Results

### Water Physico-Chemical Analysis

As shown in Supplementary Table 4, water temperature was stable over the duration of each experiment, keeping values close to 16 and 24°C for both tests respectively. pH values ranged between 6.6 and 7.6 during the 30 days of the biofilm growth phase, and no statistical differences were detected between temperatures (*P*-value >0.05). A higher concentration of total chlorine (average at 16°C = 0.93 mg/L, average at 24°C = 0.80 mg/L, *n* = 3) and free chlorine (average at 16°C = 0.89 mg/L, average at 24°C = 0.68 mg/L, *n* = 3) was observed at both temperatures on day 0, associated with the process of disinfection (i.e., hyperchlorination) of the system at the beginning of the experiment. Then, during the growth phase and after the mobilisation phase, average chlorine concentrations showed higher values at 16°C (total chlorine average = 0.19 mg/L, *n* = 12; free chlorine average = 0.13 mg/L, *n* = 12) than at 24°C (total chlorine average = 0.15 mg/L, *n* = 12; free chlorine average = 0.09 mg/L, *n* = 12), but no statistical differences were found (*p*-value >0.05). TOC, iron and manganese concentrations were similar showing no statistical differences (*p*-values >0.05) during the growth phase at both temperatures, ranging from 1.13–1.35 mg/L, 42.4–59.3 μg/L and 0.33–0.52 μg/L respectively. Online turbidity measurements during the growth phase, showed similar values at both temperatures, average of 0.043 ± 0.01 NTU at 16°C (*n* = 43181) and 0.042 ± 0.01 NTU at 24°C (*n* = 43173) (*p*-value >0.05).

**FIGURE 1:**
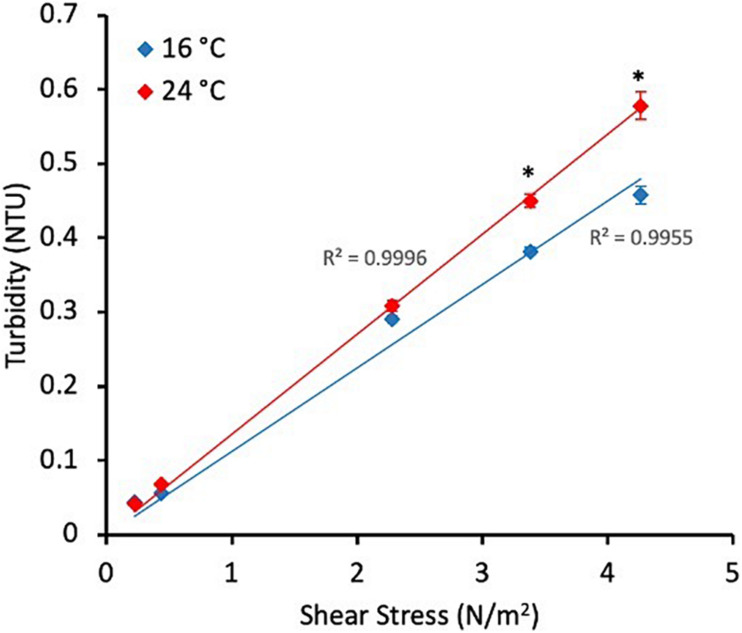
Average turbidity response during the last 24 h of the growth phase (representative of the growth phase) and during the last turnover of each stage of the flushes (i.e., when the water with the mobilised material was mixed) at different temperatures. All values represent an average ± standard deviation. ^∗^*p*-value ≤0.05 from Mann–Whitney *U* test.

After the 30 days biofilm-growth phase, shear stress was increased in the system and the physico-chemistry of the bulk water monitored to quantify the response. Fe and Mn concentration increased considerably after the mobilisation phases at both temperatures, showing significantly higher values at 24°C (Fe average = 81.2 μg/L, *n* = 3, Mn average = 1.2 μg/L, *n* = 3) than at 16°C (Fe average = 53.7 μg/L, *n* = 3; Mn average = 0.69 μg/L, *n* = 3) (*p*-value Fe = 0.03, *p*-value Mn = 0.05). The online turbidity data from the final well mixed turnover of the system after each increase in shear stress was averaged and plotted against shear stress to observe the strength profiles of the material mobilised (Figure 1). Statistically no differences were found (*p*-value >0.05) at the first two small increase in shear stress due to the relatively small amounts of material mobilised. In the last two stages statistically significant higher values in the turbidity response were observed at 24°C (*p*-value S3 = 0.0009, *p*-value S4 = 0.0006). The response at both temperatures is linear between imposed shear stress and turbidity, R^2^ = 0.9955 at 16°C and R^2^ = 0.9996 at 24°C (Figure 1).

### Scanning Electron Microscope Micrographs

Supplementary Figure 3 shows SEM micrographs of biofilm on the coupons on day 0 (control) and after 30 days of growth phase at 16 and 24°C. Sterilised coupons at the beginning of the experiment showed no cells attached to the surface of the coupon at 16 and 24°C respectively (Supplementary Figure 3A). Differences in the coupons surface coverage can be observed on day 30 at different temperatures (Supplementary Figures 3B,C). At 16°C small patches of biofilm-like structures were developed on the surface of the coupon. However, at 24°C a greater amount of cellular material and biofilm accumulation was observed on the surface of the coupon. More angular inclusions, indicative of inorganics, are visible in the 24°C than 16°C images.

### Microbial Community Structure (Bacteria and Fungi) During Growth Phase and Mobilisation

#### Alpha-Diversity (Diversity Within Samples)

**FIGURE 2:**
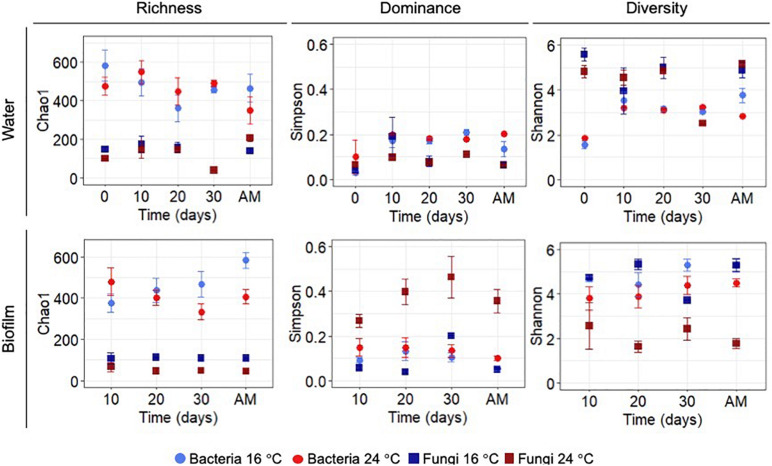
Chao 1 (richness), Simpson (dominance) and Shannon (diversity) indices for OTUs at 97% cut-off for bacteria and fungi in biofilm and water samples calculated for each sampling day during the growth phase and after mobilisation phase (AM). All values represent an average of three water replicates ± standard deviation.

Statistical differences between temperature treatments were examined by pairwise comparisons for each sampling day during the growth phase (i.e., day 0, 10, 20, and 30). Figure 2 shows the diversity indices, Chao 1, Simpson and Shannon at OTUs level at 97% cut-off for bacteria and fungi. Planktonic communities during the growth phase showed no significant differences between the experiments at different temperatures (*p*-values >0.05). Alpha-diversity of the biofilm bacterial communities did not show significant differences between temperatures during the growth phase (*p*-values >0.05).

After the mobilisation phase, Chao 1 and Shannon indices showed lower significant values at higher temperature (*p*-value Chao1 = 0.05, *p*-value Shannon = 0.05). Conversely, biofilm fungal communities showed significant differences for all the calculated indices over time (*p*-value Simpson-Day 10 = 0.05, *p*-value Shannon-Day 10 = 0.05; *p*-value Chao1-Day 20 = 0.05, *p*-value Simpson-Day 20 = 0.05, *p*-value Shannon-Day 20 = 0.05; *p*-value Chao1-Day 30 = 0.05, *p*-value Simpson-Day 30 = 0.01, *p*-value Shannon-Day 30 = 0.01) with the exception of day 10 where no differences were observed for Chao 1 (*p*-value >0.05). After flushing at 24°C the Shannon index of planktonic bacterial communities decreased (*p*-value = 0.05), and Chao 1 of fungal planktonic communities increased (*p*-value = 0.05), mirroring the response in the biofilm. See Supplementary Table 5 for more details on the results of all pairwise comparisons.

#### Beta-Diversity (Diversity Among/Between Samples)

**FIGURE 3:**
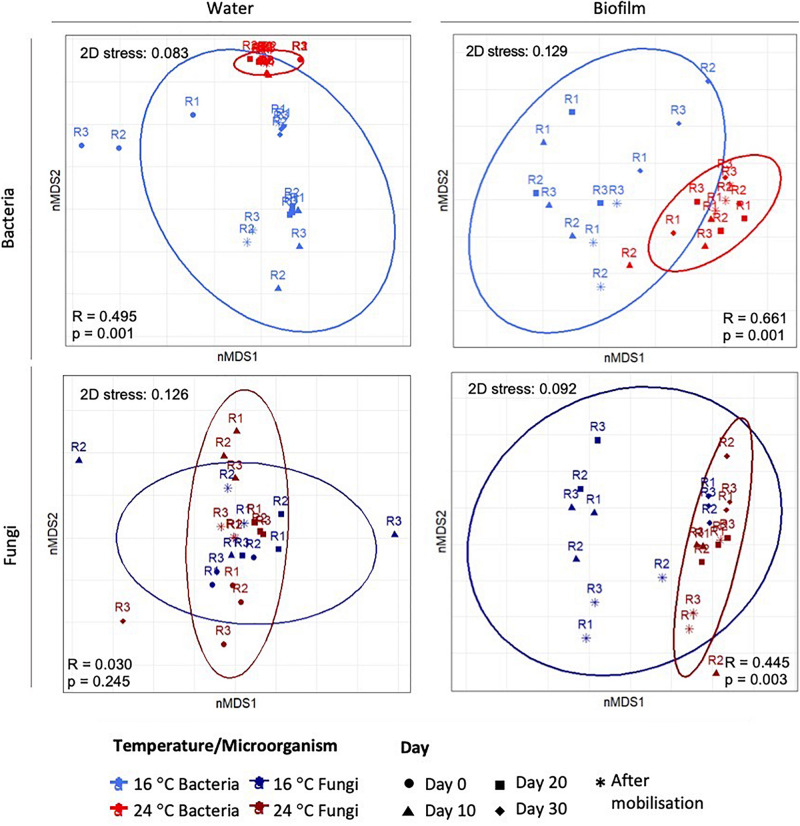
Two-dimensional plot of the non-multidimensional scaling (nMDS) analysis based on Bray–Curtis similarities of the relative abundance of bacteria and fungi in water and biofilm samples. The 3 replicates (R1, R2, and R3) per sampling day are represented.

Figure 3 shows nMDS plots with the resemblance between microbial communities at OTUs level at 97% cut-off at different temperatures. For planktonic communities, nMDS showed a clear separation for bacteria, but not for fungi. ANOSIM analysis for water samples showed that only bacterial structure was significantly modified by the increase of temperature (global-R = 0.5, *p*-value = 0.001), while fungal communities showed no significant differences between temperatures (global-R = 0.03, *p*-value = 0.245). For biofilm, nMDS showed an evident clustering at different temperatures for both, bacteria and fungi. ANOSIM confirmed that the temperature increases significantly affected the bacterial (global-R = 0.66, *p*-value = 0.001) and fungal community structures (global-R = 0.45, *p*-value = 0.003).

### Microbial Taxonomic Profiles

**FIGURE 4:**
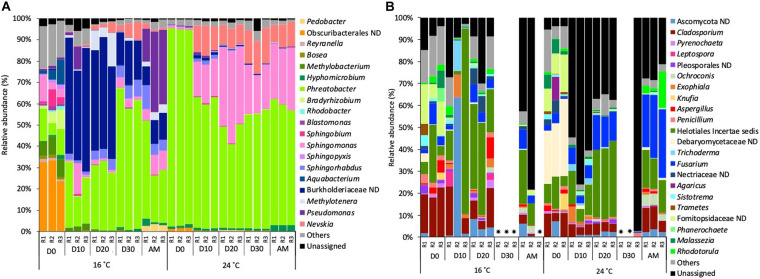
Relative abundance of **(A)** bacterial genera and **(B)** fungal genera (>1% of the total sequences) at 16 and 24°C in bulk water samples every 10 days (D) throughout the growth phase and after the mobilisation phase (AM). The 3 replicates (R1, R2, and R3) per sampling point are represented. Remaining genera were combined in category “Others.” Category “Unassigned” correspond to unidentified OTUs and “ND” means not defined at that level. 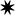 Samples that did not amplify during the sequencing process.

**FIGURE 5:**
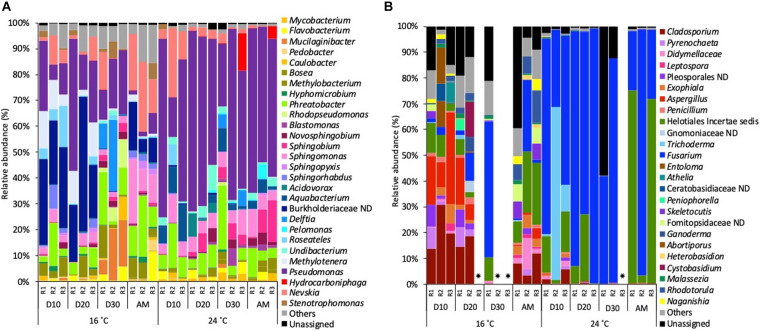
Relative abundance of **(A)** bacterial genera and **(B)** fungal genera (>1% of the total sequences) at 16 and 24°C in biofilm samples every 10 days (D) throughout the growth phase and after the mobilisation phase (AM). The 3 replicates (R1, R2, and R3) per sampling point are represented. Remaining genera were combined in category “Others.” Category “Unassigned” correspond to unidentified OTUs and “ND” means not defined at that level. 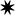 Samples that did not amplify during the sequencing process.

Focussing on the most abundant taxa (relative abundance >1%), several differences between temperatures were detected in the taxonomical composition in both water and biofilm samples at OTUs level at 97% cut-off (Figures 4, 5).

Bacterial planktonic communities, at 16°C on day 0 bacterial communities were dominated by an uncultured Obscuribacterales (30.2%) followed by *Phreatobacter* (11.2%). On consecutive days, Burkholderiaceae (39.2%) and *Phreatobacter* (37.8%) were the most abundant taxa. After the mobilisation phase, *Phreatobacter* (32.4%) dominated the community followed by *Pseudomonas* (30.0%) and a not defined (ND) Burkholderiaceae (11.1%). At 24°C planktonic communities showed a similar taxonomic profile during the growth phase and after the mobilisation, being *Phreatobacter* (61.8%) the most abundant genus, followed by *Sphingomonas* (28.5%) and *Nevskia* (9.5%) (Figure 4A).

Planktonic fungal communities showed a high percentage of unassigned sequences (ranged from 8.4 to 97.1%) in all the samples, particularly at 24°C. Overall, similar taxonomical composition was observed at 16°C and 24°C during the growth phase. After mobilisation and community was dominated by Helotiales *incertae sedis* (20.6%), *Cladosporium* (average 8.5%) and an unidentified Ascomycota (4.9%) (Figure 4B).

Biofilm communities, at 16°C, on days 10 and 20 bacterial community was dominated by a not defined (ND) Burkholderiaceae (average of 30.9%), followed by *Pseudomonas* (20.8%) and *Methylotenera* (9.2%). On day 30, *Pseudomonas* (18.5%) became the most abundant genera, followed by *Mucilaginibacter* (14.5%) and *Delftia* (12.4%). After the mobilisation phase, biofilm bacterial community was dominated by *Nevskia* (20.6%), *Sphingomonas* (14.7%) and *Phreatobacter* (14.2%). At 24°C the most representative genera at each sampled point of the growth phase was *Pseudomonas* (52.0%) followed by *Phreatobacter* (7.1%) and *Sphingomonas* (6.2%). After the mobilisation phase, *Pseudomonas* (56.5%) continued being the most abundant genus, followed by *Sphingobium* (8.6%) and *Sphingomonas* (6.6%) (Figure 5A).

For biofilm fungal communities at 16°C on days 10 and 20 *Cladosporium* (19.0%) and *Aspergillus* (average of 16.9%) were the most abundant taxa. On day 30, the relative abundance of *Fusarium* increased to become the most abundant genus (52.7%) followed by Helotiales *incertae sedis* (9.1%). After the mobilisation phase, Helotiales *incertae sedis* (18.8%), *Fusarium* (9.8%) and *Rhodotorula* (8.54%) dominated the community. At 24°C, *Fusarium* (68.6%) was the most abundant taxa over the biofilm growth phase, followed by *Trichoderma* (26.1%) and Helotiales *incertae sedis* (10.8%). After the mobilisation phase, Helotiales *incertae sedis* (49.8%) and *Fusarium* (48.3%) continued being the most abundant taxa (Figure 5B).

### Opportunistic Pathogens Occurrence Analysed by q-PCR

The OPs gene copies number at 16 and 24°C are shown in Table 1. The majority of OP selected showed no statistically significant difference between the different temperatures for water or biofilm samples. The results did show statistical differences between temperatures for *Mycobacterium* spp. on biofilm samples from day 30 (*p*-value ≤0.05). However, in the rest of the biofilm samples and in water samples this microorganism did not show statistical differences. The gene copy number for *M. avium* complex showed statistical changes between temperatures; at 16°C the gene copies were below the limit of quantification, but they showed an increase at 24°C in all the samples analysed (biofilm and water). *P. aeruginosa*, *Acanthomoeba* spp. and *S. maltophilia* were detected and quantified in all samples of biofilm and water over time (except *Acanthomaeba* spp. after the mobilisation phase in both water and biofilm). *L. pneumophillia* was below quantification limit in every sampling point. Overall, the OPs gene copy number in biofilm samples tended to increase at 24°C (with 14 out of 19 relevant cases, but with only 5 statistically significant), yet not a clear pattern was observed with biofilm age. *L. pneumophillia* was below quantification limit in every sampling point at 16 and 24°C.

**TABLE 1:** Occurrence of *Mycobacterium* spp., *M. avium* complex, *P. aeruginosa*, *L. pneumophillia*, *Acanthamoeba* spp. and S. *malthophilia* in biofilm and water samples at 16 and 24°C.

		*Mycobacterium spp.*		*M. avium* complex		*P. aeruginosa*	
		16°C	24°C	16°C	24°C	16°C	24°C
Biofilm (copies/cm^2^)	Day 10	4.99E + 02 ± 2.48E + 02	9.02E + 02 ± 1.26E + 02	B.Q.L	1.94E + 02 ± 2.91E + 01 *	2.32E + 03 ± 3.33E + 02	1.38E + 03 ± 5.85E + 00
	Day 20	1.60E + 03 ± 8.19E + 02	7.77E + 03 ± 5.5E + 03	B.Q.L	3.12E + 02 ± 9.21E + 00 *	4.95E + 02 ± 3.21E + 02	7.06E + 02 ± 1.29E + 01
	Day 30	1.90E + 03 ± 1.23E + 03	1.14E + 04 ± 2.82E + 03 *	B.Q.L	2.17E + 02 ± 8.22E + 01 *	7.53E + 02 ± 2.33E + 02	1.41E + 03 ± 1.52E + 02
	AM	1.21E + 03 ± 1.02E + 03	4.37E + 03 ± 2.70E + 03	B.Q.L.	3.70E + 02 ± 5.24E + 01 *	1.25E + 03 ± 1.28E + 02	1.46E + 03 ± 9.14E + 01
Water (copies/L)	Day 0	7.59E + 03 ± 4.12E + 03	2.42E + 03 ± 3.97E + 02	B.Q.L.	1.14E + 02 ± 7.00E + 01 *	1.95E02 ± 1.02E + 02	7.18E + 02 ± 2.27E + 02
	Day 10	2.05E + 04 ± 7.31E + 02	1.73E + 04 ± 2.59E + 03	B.Q.L.	2.94E + 02 ± 4.77E + 01 *	3.23E + 02 ± 4.45E + 01	1.50E + 03 ± 4.89E + 02
	Day 20	1.02E + 04 ± 3.20E + 03	9.55E + 03 ± 3.76E + 03	B.Q.L.	2.20E + 02 ± 5.47E + 01 *	3.21E + 02 ± 1.92E + 02	2.45E + 03 ± 7.79E + 02
	Day 30	1.68E + 04 ± 1.31E + 03	1.42E + 04 ± 2.34E + 02	B.Q.L.	3.54E + 02 ± 8.23E + 01 *	3.18E + 03 ± 1.23E + 03	2.81E + 03 ± 3.44E + 02
	AM	2.09E + 04 ± 7.23E + 03	3.85E + 04 ± 3.40E + 03	B.Q.L.	2.43E + 02 ± 1.02E + 02 *	4.73E + 03 ± 1.84E + 03	3.10E + 03 ± 1.15E + 03

		***L. pneumophillia***		***Acanthomaeba spp.***		***S. maltophilia***	
		**16°C**	**24°C**	**16°C**	**24°C**	**16°C**	**24°C**

Biofilm (copies/cm^2^)	Day 10	B.Q.L	B.Q.L	1.74E + 04 ± 5.71E + 03	1.85E + 04 ± 5.23E + 03	2.17E + 03 ± 1.20E + 03	1.5E + 03 ± 1.16E + 03
	Day 20	B.Q.L	B.Q.L	1.21E + 04 ± 3.64E + 03	2.14E + 04 ± 8.03E + 03	1.81E + 03 ± 3.04E + 02	1.95E + 03 ± 1.91E + 02
	Day 30	B.Q.L	B.Q.L	7.89E + 03 ± 1.58E + 03	2.18E + 04 ± 8.18E + 03	3.09E + 03 ± 1.35E + 03	1.88E + 03 ± 7.61E + 02
	AM	B.Q.L	B.Q.L	B.Q.L.	B.Q.D.	2.84E + 03 ± 7.84E + 01	9.60E + 02 ± 7.30E + 02
Water (copies/L)	Day 0	B.Q.L.	B.Q.L.	1.86E + 04 ± 2.09E + 03	1.18E + 04 ± 6.46E + 03	1.38E + 03 ± 1.09E + 03	1.91E + 03 ± 1.39E + 03
	Day 10	B.Q.L	B.Q.L	1.73E + 04 ± 7.85E + 03	2.04E + 04 ± 4.77E + 03	7.32E + 03 ± 2.24E + 03	4.54E + 03 ± 2.14E + 03
	Day 20	B.Q.L	B.Q.L	2.06E + 04 ± 2.17E + 03	3.30E + 04 ± 1.32E + 04	4.44E + 03 ± 2.37E + 03	6.96E + 03 ± 2.46E + 03
	Day 30	B.Q.L	B.Q.L	1.50E + 04 ± 2.31E + 03	1.66E + 04 ± 4.00E + 03	4.28E + 03 ± 1.86E + 03	3.29E + 03 ± 1.28E + 03
	AM	B.Q.L	B.Q.L	2.55E + 04 ± 4.10E + 03	4.73E + 04 ± 1.43E + 04	8.64E + 02 ± 0.00E + 00	0.00E + 00 ± 0.00E + 00

All values represent an average of three replicates ± standard deviation. “B.Q.L” correspond to number of genes below quantification limit. *p-value ≤0.05 from Mann–Whitney *U* test.

The water samples showed no statistically significant change with time or between repeats, again confirming the stability of the incoming water and that temperature was the dominant environmental change between the repeat experiments.

## Discussion

### Effect of Temperature Rise on Water Physico-Chemistry and Discolouration

Results showed that most water physico-chemical characteristics during the growth phase were stable over time (Supplementary Table 4), and most importantly comparable at both temperatures such that temperature was the dominant variable between the experiments.

Free and total chlorine showed slightly lower concentrations during the growth phase at 24°C. Although the differences were not significant between temperatures, this is consistent with the understanding that increasing temperature increases reaction rates. The level of disinfectant residual plays an important role in DWDS by limiting the microbial growth and preventing the proliferation of pathogenic microorganisms (Donnermair and Blatchley, 2003; Fisher et al., 2012; Karadirek et al., 2016). Hence in the scenario of increasing temperature, and with operational systems residence times being greater than the average 24 h of the study system, maybe it will be necessary to augment the doses of disinfectant or look for alternative disinfectants that do not use chlorine-based compounds. Thus, future increases in temperature will pose an economic and technical challenge for water utilities to maintain disinfectant residual concentrations throughout the entire distribution network without compromising the health of consumers.

When the process of water discolouration was evaluated, results showed similar turbidity values and Fe and Mn concentrations during the growth phase at 16 and 24°C, however, there was marked difference in response to biofilm mobilisation (Supplementary Table 4 and Figure 1). Consistency in water turbidity and Fe and Mn concentrations was expected during the growth phase, confirming minimal changes in the source water source during and between the experiments. Discolouration events are related to the biofilm mobilisation from the pipe wall, which is normally produced by a change in the hydraulic conditions (Vreeburg and Boxall, 2007). During the growth phase, the hydraulic conditions of the system were constant following a daily pattern of LVF, explaining the expected absence of changes in turbidity and metal concentrations. After the mobilisation phase, the concentration of these metals and turbidity response increased significantly at both temperatures with significant higher values for these two parameters observed after the flushing event at 24°C. These results indicate that higher temperature led to a greater material accumulation on this chlorinated DWDS, as it was observed by Blokker and Schaap (2015) in unchlorinated DWDS. This finding is in agreement with the SEM micrographs obtained from coupons, which showed a visual greater accumulation of biofilm on the surface of the coupon at 24°C. Previous research, reported a link between microbial growth and temperatures; Hallam et al. (2001) concluded that biofilm potential growth in chlorinated DWDS was enhanced at higher temperatures; and Ahmad et al. (2020) observed higher biofilm concentrations in unchlorinated DWDS with cold recovery technologies concluding that microbial growth kinetics in biofilms were affected by temperature. Thus, the increase in growth and microbial activity in DWDS biofilms due to temperature rise may lead to a greater amount of EPS production, which might facilitate the adsorption and entrainment of material from the bulk water (Douterelo et al., 2019) and the release of OPs into the bulk water.

Importantly, our research also looked at the effects of temperature on the strength profile of the material accumulations. We used a stepped flushing regime such that we could observe the amount of material mobilised by each imposed force in turn. The function of cumulative release of material due to these sequential increases in imposed force was linear (Figure 1). This shows that there was not preferential growth/accumulation of stronger or weaker material as a function of temperature. However, the gradient of the trend was greater for the higher temperature regime, showing greater material accumulation across all the strengths of the cohesive biofilm mediated layers. In brief, under the increased temperature more material was accumulated and subsequently mobilised across all strengths of the biofilm layers, there was no preferential accumulation in stronger or weaker layers.

Our results show that higher temperatures in chlorinated DWDS promote biofilm accumulation on HDPE pipe walls and that when the material attached to the pipe walls is mobilised the probability and severity for water discolouration is greater. We also observed that the strength characteristics of the biofilm layers are unchanged, suggesting existing modelling and intervention strategies (Husband and Boxall, 2011; Husband et al., 2016) remain valid, but that the frequency of these would need to be increased to mitigate the accelerated effects.

### Influence of Temperature Upon Microbial Community Structure and Taxonomical Profiles

In addition to the greater potential for biofilm formation at high temperatures, molecular analyses showed that temperature variation significantly modified the structure of biofilm microbial communities from the early stages of biofilm development (Figures 2, 3). This was in agreement with Stanish et al. (2016) that reported that water temperature was determinant for the abundance and composition of bacterial communities in tap water samples, or with Ahmad et al. (2020) and Zhou et al. (2020) that showed that higher temperatures promoted by cold recovery technologies, in unchlorinated and chlorinated DWDS respectively, produced changes in biofilm communities. In the same way, the results from this study confirm that temperature increase is a driving factor changing the bacterial but also fungal microbial community structure within chlorinated DWDS. Furthermore, it has been shown that these differences appear from the early stages of biofilm development, developed at constant temperatures and under representative hydraulic conditions.

These structural changes were evident when the taxonomical composition was analysed over time (Figures 4, 5). Regarding bacteria (Figure 4A), taxa such as Burkholderiaceae ND, *Methylotenera* or *Methylobacterium*, decreased in relative abundance at 24°C. The reduction of Burkholderiaceae could be beneficial for water quality, since members of this family have been reported as one of the most common contaminants in distilled and sterile water and as phyto- and animal and human pathogen (Coenye, 2014; Ferranti et al., 2014). In contrast, the lower relative abundance of *Methylotenera* and *Methylobacterium* at higher temperature does not suggest positive effects for water quality. Tsagkari and Sloan (2019) demonstrated that *Methylobacterium*, even at low concentrations, is able to decrease the concentration of trihalomethanes in drinking water (World Health Organization (WHO), 2017; Tsagkari and Sloan, 2019). *Methylotenera* have been attributed to play an important role in methanol-linked denitrification in lake sediments (Kalyuhznaya et al., 2009), and is able to biodegrade nitrite in water, compound that is often associated with the formation of harmful DBPs in DWDS (Rantanen et al., 2018). Therefore, their lower relative abundance can affect the positive biodegradation capabilities of these microorganisms. Other important genus, *Pseudomonas*, increased its abundance in biofilms developed at 24°C. Its presence was expected at both temperatures since they often appear in DWDS independently of the pipe material, source water or hydraulic conditions (Martiny et al., 2005; Zhang et al., 2012; Douterelo et al., 2016). However, the increase of *Pseudomonas* could have negative consequences for water safety, since several opportunistic pathogens belong to this genus (Zhang et al., 2012; Liu et al., 2016). In addition, *Pseudomonas* spp. are pioneers in the initial stages of biofilm development since they are capable of secreting a high amount of EPS that enhance the growth of biofilms (Johnsen et al., 2000; Simões et al., 2007; Irie et al., 2012; Navarro-Noya et al., 2013). The increase of this genus could lead to an increase in EPS production, which reinforces the hypothesis described above about the greater biofilm formation at higher temperatures. Moreover, EPS can act as protection for the cells (Flemming, 2002), and the potential larger production at higher temperatures would make biofilm communities more resistant against changes in the environmental pipe conditions including disinfectants. This would explain that the microbiome composition during the growth phase and after mobilisation were more similar to each other at 24°C than at 16°C.

Temperature also clearly influenced the fungal community structure in biofilms (Figure 4B). In general, alpha-diversity of biofilm fungal communities were more affected by temperature changes than bacteria during the studied biofilm-growth phase, showing the loss of diversity and richness, and the increase in dominance. This is in agreement with Ortiz-Vera et al. (2018) who found that fungal dominance and diversity in rivers were affected by temperature. To the best of our knowledge this study shows for the first time a similar behaviour for fungal communities in chlorinated DWDS, confirming that these microorganisms are an important part of the biological component of DWDS. The fungal genus that dominated the biofilm community, displacing other microorganisms at higher temperatures was *Fusarium*. The optimal growth temperature of several species of these filamentous fungi range from 24.7 to 27.5°C (Nazari et al., 2018), which would explain its proliferation at 24°C. *Fusarium* is able to produce EPS, thus enhancing biofilm formation (Anaissie et al., 2001; Sav et al., 2018), and as suggested for *Pseudomonas*, its high relative abundance could contribute to the greater biofilm formation observed at 24°C. In addition, several pathogenic species belonging to this genus produce nosocomial infections that mainly affect immunocompromised patients (Anaissie et al., 2001). Therefore, the high relative abundance of these genera into the bulk water enhanced by higher temperature is not desirable, since it could lead to water quality and safety problems.

Temperature variation had a lower effect on bulk water samples (Figure 5). From day 0, the water already had different temperatures, and this explain the different microbial composition on Day 0 for both experimental setups. Then, during the growth phase, temperature effect in planktonic communities was mainly limited to increases in the relative abundance of several genera including *Phreatobacter* and *Sphingomonas* at 24°C. These results suggest that water communities, which are more affected and controlled by disinfection processes, are less likely to be modified by other factors such as temperature (Simões et al., 2010). However, biofilms provide microorganisms physical and chemical protection, and other physiological advantages generating a more favourable environment over planktonic cells (Flemming, 2002). Therefore, as biofilms are more resistant to disinfectants, this makes them more susceptible to being affected by other abiotic factors such as temperature. Regarding the genera that increased their abundance, *Phreatobacter* was recently isolated from ultrapure water of a water purification system (Toth et al., 2014), and it has not been reported by other studies, thus its ecological role in many ecosystems remains unknown. *Sphingomonas* species are well adapted to oligotrophic environments and they are known for its potential to form biofilms in drinking water systems (Gulati and Ghosh, 2017). However, during the growth phase the system was refreshed with incoming water every 24 h, thus planktonic communities are renewed and not representative of communities growing constantly at 24°C.

### Occurrence of OPs

Results showed that *P. aeruginosa*, *S. maltophilia* and *Acanthamoeba spp*. were detected in practically all biofilm and water samples when analysed using q-PCR. Differences in the gene copy number of these OPs were observed at 16 and 24°C, however, statistical analysis showed that they were not significant (Table 1). Similar values for these particular OPs have been reported previously in unchlorinated systems at temperatures ranging from 8.7 to 14.8°C (van der Wielen and van der Kooij, 2013), but these results show them able to survive in chlorinated system at 16 and 24°C. In addition to these OPs, *Mycobacterium* spp. was present in all samples analysed in this study, higher gene copy numbers in all biofilm samples at 24°C compared to 16°C were quantified although only statistically significantly at day 30. The presence of this OP has been linked to important nosocomial respiratory infections (Liu et al., 2016), therefore its higher occurrence with the temperature rise is a concern. There was no quantification of *M. avium* complex at 16°C in biofilm or water samples, but this pathogen was detected in all samples at 24°C. This is in agreement with the findings of Daley (2017) who reported that temperature impacted the relative abundance of *M*. *avium* complex in tap water samples from a pilot-scale DWDS at temperatures between 39 and 51°C. However, our results extend this showing that *M. avium* also depends on temperature in biofilms within chlorinated DWDS. In addition, it has been shown that less drastic changes in temperature can increase the abundance of *M. avium* that can cause pulmonary or even disseminated infections in immunocompromised patients (Daley, 2017). Moreover, since the occurrence of *Mycobacterium spp*. could not be explained only with the detection of *M. avium* complex, these results suggest that other species of mycobacteria are present in the samples analysed in our study. Further research is needed to identify these species and have a full picture about the OPs presence and activity and their potential release from biofilms into the bulk water.

## Conclusion

• Higher temperatures increased biofilm accumulation on HDPE pipes in a chlorinated DWDS, leading to a higher discolouration (quantified as turbidity, iron and manganese) due to simulated flushing. The mobilisation of this material was a linear function with imposed force, showing that while material accumulation was accelerated by temperature, it was not preferentially to either stronger or weaker biofilm layers.
• Temperature triggered changes in the microbiome of the DWDS. Temperature affected the structure of bacterial and fungal communities in water, but changes were more notable in biofilms. In biofilms, the relative abundance of *Pseudomonas* and *Fusarium* was favoured by temperature increase. These two genera have an enhanced capability to promote biofilm development and compromising further water quality.
• The study of OPs showed that temperature rise increased the detection of *Mycobacterium* spp. in biofilms and favour the presence of *M. avium* complex in water and biofilm samples.

This study provides new insights on the consequences of higher temperatures on the distribution of drinking water and its impact on water quality. This research is essential to mitigate risks and adapt this fundamental transport infrastructure to prevent the effects of unavoidable climate change.

## Data Availability Statement

The datasets presented in this study can be found in online repositories. The names of the repository/repositories and accession number(s) can be found below: https://www.ncbi.nlm.nih.gov/, PRJNA656259.

## Author Contributions

CC, JB, and ID involved in the design of the experiment. CC and ID were in charge of the adaptation of the manuscript for a journal publication and carried out the experiment. CC performed the DNA extraction from samples, analysed the results and did the SEM micrographs, and wrote the manuscript. CC, VS-C, and SM involved in the bioinformatic analysis. CC, JB, VS-C, SM, and ID contributed to the interpretation of results. JB and ID participated in the corrections of the manuscript. All authors contributed to the article and approved the submitted version.

## Conflict of Interest

The authors declare that the research was conducted in the absence of any commercial or financial relationships that could be construed as a potential conflict of interest.

## Supplementary Material

Supplementary Figure 1 **(A)** Full scale experimental DWDS facility; **(B)** Coupons inserted and fixed into the pipes with holders and clips; **(C)** details and dimensions of the coupons: insert for microscopy and outer for DNA analysis.

Supplementary Figure 2 Flushing gradual steps (S) applied in this research for a duration of 3 turnovers.

Supplementary Figure 3 SEM micrographs of biofilm developed in coupons: **(A)** Control (sterile coupon) (MAG = 5.00 kx); **(B)** Day 30 at 16°C (MAG = 5.00 kx); **(C)** Day 30 at 24°C (MAG = 5.00 kx). MAG, magnification.

Supplementary Table 1 Test loop facility phases flow rate conversions.

Supplementary Table 2 Sequence count during each step of bioinformatic analysis of the bacterial 16S rRNA gene in all biofilm and water samples at 16 and 24°C. (-) Samples that did not amplify or were excluded during the analysis for not having enough readings.

Supplementary Table 3 Sequence count during each step of bioinformatic analysis of the fungal ITS1-2 region in all biofilm and water samples at 16 and 24°C. (-) Samples that did not amplify or were excluded during the analysis for not having enough readings.

Supplementary Table 4 Water physico-chemical parameters measured on sampling days during the growth phase and after mobilisation phase (AM). All values represent an average of three water replicates analysis ± standard deviation.

Supplementary Table 5 Results from the Mann-Whitney *U* test to determine statistical differences in bacterial and fungal alpha diversity indices between temperatures. Differences were considered statistically significant when *p*-value was ≤0.05.
