# Supplementary figures and images for: Implications of Climate Change: How Does Increased Water Temperature Influence Biofilm and Water Quality of Chlorinated Drinking Water Distribution Systems?
Source: Front Microbiol. 2021 Jun 8;12:658927. doi: 10.3389/fmicb.2021.658927 (PMC8217620; doi:10.3389/fmicb.2021.658927)

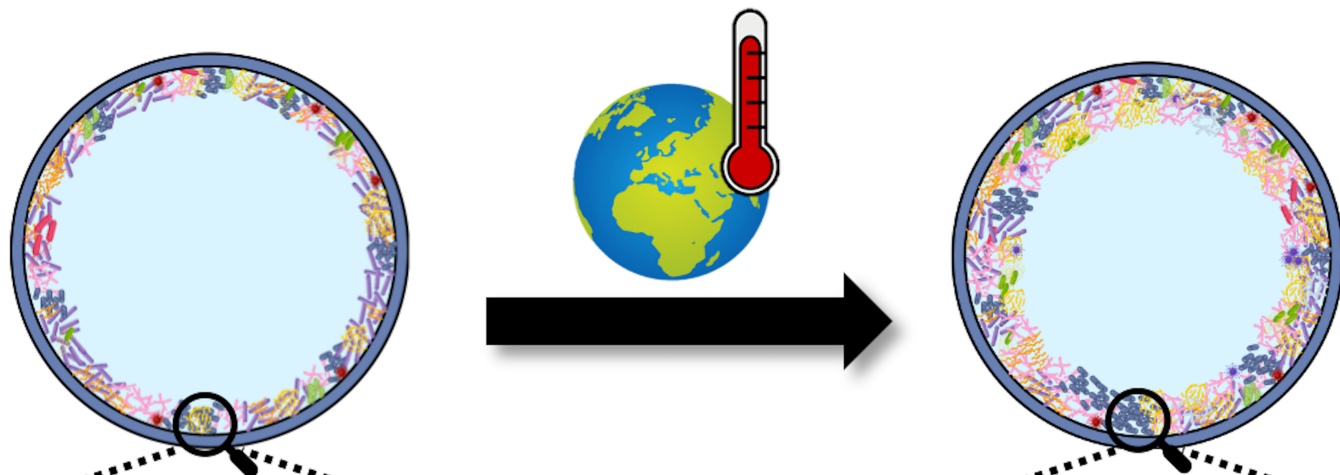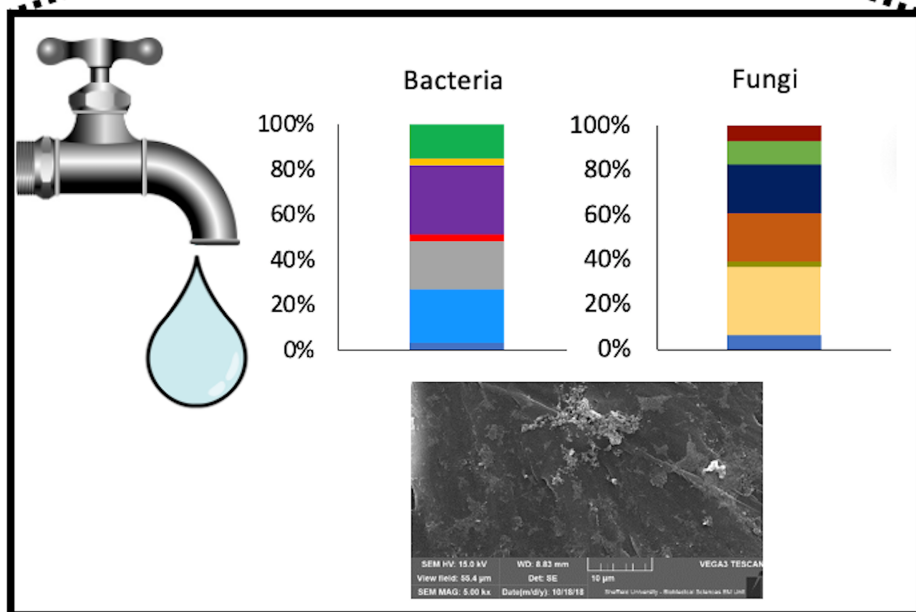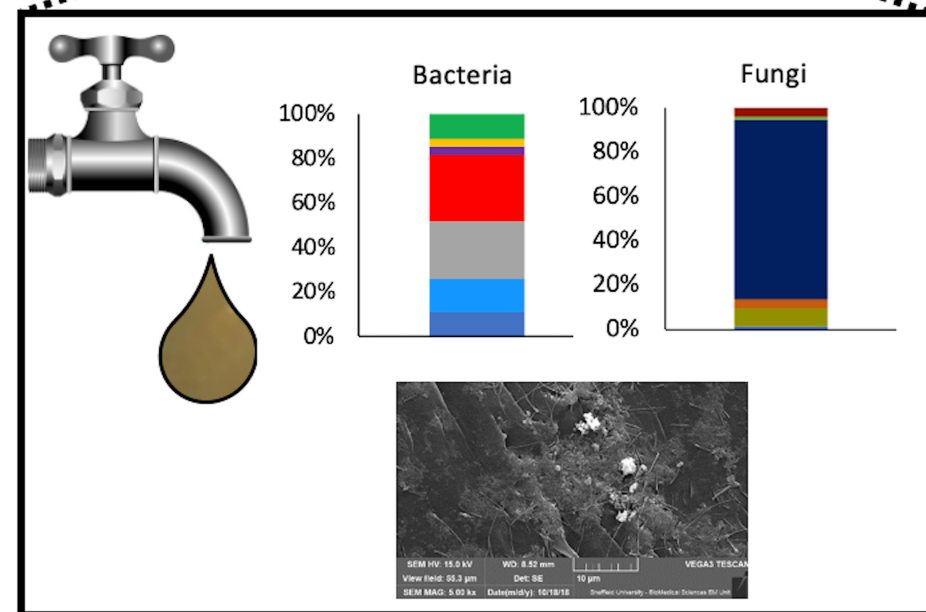

Supplement: Supplementary file 1 [file Presentation_1.PDF]

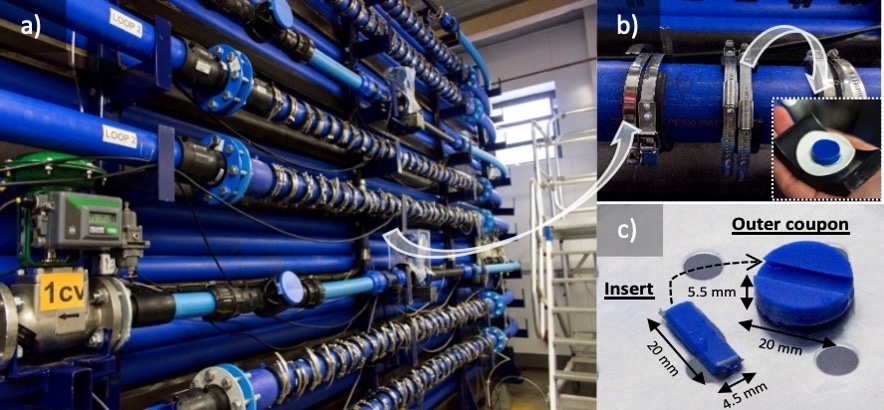

Supplement: Supplementary Figure 1 — (A) Full scale experimental DWDS facility; (B) Coupons inserted and fixed into the pipes with holders and clips; (C) details and dimensions of the coupons: insert for microscopy and outer for DNA analysis. [file Image_1.JPEG]

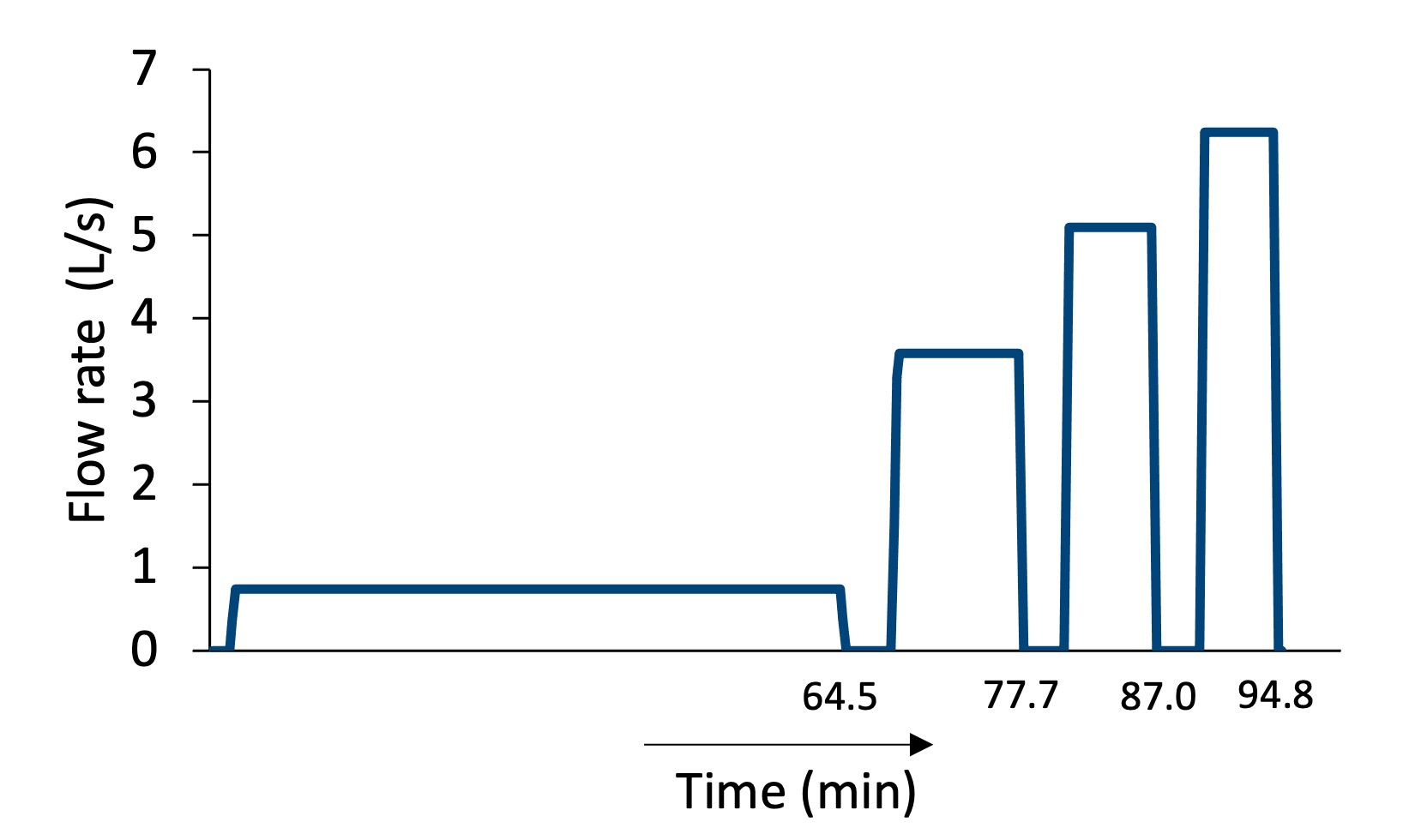

Supplement: Supplementary Figure 2 — Flushing gradual steps (S) applied in this research for a duration of 3 turnovers. [file Image_2.JPEG]

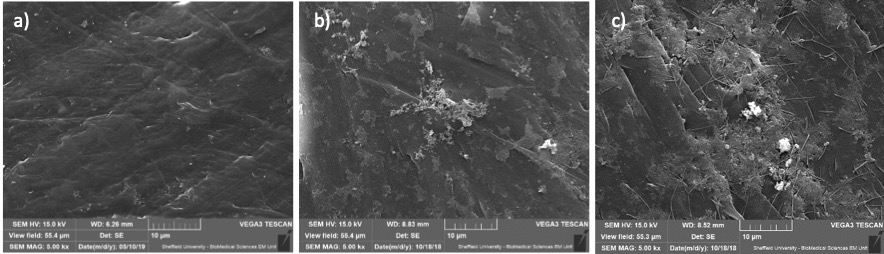

Supplement: Supplementary Figure 3 — SEM micrographs of biofilm developed in coupons: (A) Control (sterile coupon) (MAG = 5.00 kx); (B) Day 30 at 16°C (MAG = 5.00 kx); (C) Day 30 at 24°C (MAG = 5.00 kx). MAG, magnification. [file Image_3.JPEG]
