# Supplementary material for: Implications of Climate Change: How Does Increased Water Temperature Influence Biofilm and Water Quality of Chlorinated Drinking Water Distribution Systems?
Source: Front Microbiol. 2021 Jun 8;12:658927. doi: 10.3389/fmicb.2021.658927 (PMC8217620; doi:10.3389/fmicb.2021.658927)
Supplement: Supplementary Table 1 — Test loop facility phases flow rate conversions. [file Table_1.PDF]

	Flow (L/s)	Velocity (m/s)	Shear stress (N/m^2)
Growth phase	0.2 - 0.5	0.04 – 0.10	0.1 – 0.3
Step 1 Mobilisation phase	0.74	0.15	0.4
Step 2 Mobilisation phase	3.58	0.72	2.3
Step 3 Mobilisation phase	5.10	1.03	3.4
Step 4 Mobilisation phase	6.24	1.27	4.3
