# Supplementary material for: Implications of Climate Change: How Does Increased Water Temperature Influence Biofilm and Water Quality of Chlorinated Drinking Water Distribution Systems?
Source: Front Microbiol. 2021 Jun 8;12:658927. doi: 10.3389/fmicb.2021.658927 (PMC8217620; doi:10.3389/fmicb.2021.658927)
Supplement: Supplementary Table 2 — Sequence count during each step of bioinformatic analysis of the bacterial 16S rRNA gene in all biofilm and water samples at 16 and 24°C. (-) Samples that did not amplify or were excluded during the analysis for not having enough readings. [file Table_2.PDF]

Sample ID	Raw reads	Filtered and imported to QIIME2	After join pair-ends	After dereplication	After clustering (97%)	After chimera filtering	Rarefied	Total OTUs observed
B 16 °C D10 R1	90654	57811	25498	25498	25498	23937	9854	329
B 16 °C D10 R2	86375	53532	26774	26774	26774	25527	9854	204
B 16 °C D10 R3	58467	25624	10383	10383	10383	9854	9854	339
B 24 °C D10 R1	119559	86716	40053	40053	40053	36681	9854	239
B 24 °C D10 R2	100450	67607	32266	32266	32266	29676	9854	257
B 24 °C D10 R3	143669	110826	48742	48742	48742	45702	9854	157
B 16 °C D20 R1	63306	30641	12533	12533	12533	11540	9854	125
B 16 °C D20 R2	100292	67627	25541	25541	25541	24157	9854	335
B 16 °C D20 R3	102204	69539	29148	29148	29148	26317	9854	377
B 24 °C D20 R1	92378	59713	21657	21657	21657	20910	9854	155
B 24 °C D20 R2	148416	115751	47890	47890	47890	44270	9854	201
B 24 °C D20 R3	123752	91087	39391	39391	39391	33719	9854	215
B 16 °C D30 R1	98498	68448	24660	24660	24660	22167	9854	393
B 16 °C D30 R2	103155	73105	21179	21179	21179	18987	9854	267
B 16 °C D30 R3	83209	53159	21081	21081	21081	18163	9854	250
B 24 °C D30 R1	63053	33003	12601	12601	12601	11873	9854	254
B 24 °C D30 R2	106780	76730	26345	26345	26345	23871	9854	204
B 24 °C D30 R3	74908	44858	13987	13987	13987	11991	9854	184
B 16 °C AF R1	101126	68461	33367	33367	33367	31267	9854	324
B 16 °C AF R2	63313	30648	15250	15250	15250	14367	9854	352
B 16 °C AF R3	86112	53447	24243	24243	24243	21931	9854	364
B 24 °C AF R1	102228	69563	28974	28974	28974	25765	9854	208
B 24 °C AF R2	98473	65808	28939	28939	28939	25458	9854	213
B 24 °C AF R3	82766	50101	22055	22055	22055	18899	9854	225
W 16 °C D0 R1	103546	58130	30835	30835	30835	29017	9854	772
W 16 °C D0 R2	108289	54253	26617	26617	26617	24741	9854	706
W 16 °C D0 R3	110607	55843	25465	25465	25465	23332	9854	581
W 24 °C D0 R1	92612	70321	45353	45353	45353	44453	9854	222
W 24 °C D0 R1	97295	78187	49330	49330	49330	48210	9854	218
W 24 °C D0 R1	113712	66268	42349	42349	42349	41441	9854	257
W 16 °C D10 R1	90973	112292	51047	51047	51047	47697	9854	180
W 16 °C D10 R2	87096	83323	39837	39837	39837	36247	9854	253
W 16 °C D10 R3	88686	207910	65752	65752	65752	61507	9854	269
W 24 °C D10 R1	103164	57199	37592	37592	37592	35435	9854	245
W 24 °C D10 R2	111030	70383	46158	46158	46158	43468	9854	257

W 24 °C D10 R3	99111	58433	38999	38999	38999	35778	9854	270
W 16 °C D20 R1	145135	118806	54499	54499	54499	50772	9854	154
W 16 °C D20 R2	116166	93391	42593	42593	42593	39880	9854	159
W 16 °C D20 R3	240753	74727	34978	34978	34978	32727	9854	160
W 24 °C D20 R1	90042	59852	38456	38456	38456	35625	9854	208
W 24 °C D20 R2	103226	69793	48328	48328	48328	43770	9854	277
W 24 °C D20 R3	91276	49698	33604	33604	33604	30819	9854	196
W 16 °C D30 R1	148856	91164	46360	46360	46360	43558	9854	160
W 16 °C D30 R2	123441	81700	37497	37497	37497	34945	9854	172
W 16 °C D30 R3	104777	95054	43827	43827	43827	41531	9854	141
W 24 °C D30 R1	89902	64826	37638	37638	37638	35232	9854	224
W 24 °C D30 R2	99843	74058	43199	43199	43199	40506	9854	202
W 24 °C D30 R3	79748	70407	41122	41122	41122	38537	9854	203
W 16 °C AF R1	121214	70881	46666	46666	46666	42260	9854	146
W 16 °C AF R2	111750	75624	40007	40007	40007	36397	9854	213
W 16 °C AF R3	125104	77942	41583	41583	41583	37595	9854	211
W 24 °C AF R1	94876	59947	41714	41714	41714	37138	9854	147
W 24 °C AF R2	104108	64630	44141	44141	44141	39938	9854	139
W 24 °C AF R3	100457	81047	54482	54482	54482	49931	9854	140
