# Supplementary material for: Implications of Climate Change: How Does Increased Water Temperature Influence Biofilm and Water Quality of Chlorinated Drinking Water Distribution Systems?
Source: Front Microbiol. 2021 Jun 8;12:658927. doi: 10.3389/fmicb.2021.658927 (PMC8217620; doi:10.3389/fmicb.2021.658927)
Supplement: Supplementary Table 3 — Sequence count during each step of bioinformatic analysis of the fungal ITS1-2 region in all biofilm and water samples at 16 and 24°C. (-) Samples that did not amplify or were excluded during the analysis for not having enough readings. [file Table_3.PDF]

Sample ID	Raw reads	Filtered and imported to QIIME2	After join pair-ends	After dereplication	After clustering (97%)	After chimera filtering	Rarefied	Total OTUs observed
B 16 °C D10 R1	33476	20363	10160	10160	10160	10138	5702	110
B 16 °C D10 R2	35243	22130	11015	11015	11015	11006	5702	92
B 16 °C D10 R3	51327	38214	19107	19107	19107	19017	5702	99
B 24 °C D10 R1	36289	18140	9140	9140	9140	9123	5702	104
B 24 °C D10 R2	66985	64876	31472	31472	31472	31096	5702	64
B 24 °C D10 R3	39603	26490	13204	13204	13204	12701	5702	102
B 16 °C D20 R1	119118	99843	49921	49921	49921	49123	5702	136
B 16 °C D20 R2	83599	64324	32110	32110	32110	32030	5702	141
B 16 °C D20 R3	-	-	-	-	-	-	-	-
B 24 °C D20 R1	82637	63362	31446	31446	31446	31276	5702	57
B 24 °C D20 R2	99175	79900	39526	39526	39526	39226	5702	69
B 24 °C D20 R3	74288	55013	27236	27236	27236	27123	5702	55
B 16 °C D30 R1	72130	50525	1195	1195	1195	1011	-	
B 16 °C D30 R2	48577	26972	482	482	482	417	-	-
B 16 °C D30 R3	50784	29179	492	492	492	435	-	-
B 24 °C D30 R1	155620	134015	6258	6257	6257	5969	5702	42
B 24 °C D30 R2	224547	202942	4016	4016	4016	3609	-	-
B 24 °C D30 R3	54948	33343	649	649	649	562	-	-
B 16 °C AF R1	127405	108130	51574	51574	51574	51299	5702	155
B 16 °C AF R2	71460	52185	25049	25049	25049	25183	5702	122
B 16 °C AF R3	107740	88465	43141	43141	43141	43983	5702	164
B 24 °C AF R1	217800	198525	93673	93673	93673	92710	5702	64
B 24 °C AF R2	72890	53615	26802	26802	26802	26673	5702	72
B 24 °C AF R3	184030	164755	80930	80930	80930	80187	5702	64
W 16 °C D0 R1	121078	101803	47311	47311	47311	47170	5702	195
W 16 °C D0 R2	88977	69702	33967	33967	33967	33741	5702	151
W 16 °C D0 R3	51542	32267	15470	15470	15470	15431	5702	149
W 24 °C D0 R1	105273	85998	41474	41474	41474	41310	5702	131
W 24 °C D0 R1	141050	121775	58559	58559	58559	58205	5702	108
W 24 °C D0 R1	119732	100457	49244	49244	49244	49111	5702	122
W 16 °C D10 R1	92193	79080	38360	38360	38360	37941	5702	256
W 16 °C D10 R2	250662	237549	117611	117611	117611	116806	5702	151
W 16 °C D10 R3	157941	144828	70127	70127	70127	69873	5702	149
W 24 °C D10 R1	63939	50826	24615	24615	24615	24562	5702	91
W 24 °C D10 R2	138901	125788	59520	59520	59520	58706	5702	232

W 24 °C D10 R3	116915	103802	49168	49168	49168	48694	5702	225
W 16 °C D20 R1	69449	56336	27550	27550	27550	27054	5702	215
W 16 °C D20 R2	66311	53198	25566	25566	25566	25247	5702	225
W 16 °C D20 R3	52672	39559	17196	17196	17196	16779	5702	209
W 24 °C D20 R1	70515	57402	27734	27734	27734	27409	5702	244
W 24 °C D20 R2	69113	56000	26014	26014	26014	25852	5702	164
W 24 °C D20 R3	90444	77331	36033	36033	36033	35403	5702	227
W 16 °C D30 R1	-	-	-	-	-	-	-	-
W 16 °C D30 R2	-	-	-	-	-	-	-	-
W 16 °C D30 R3	-	-	-	-	-	-	-	-
W 24 °C D30 R1	-	-	-	-	-	-	-	-
W 24 °C D30 R2	-	-	-	-	-	-	-	-
W 24 °C D30 R3	96667	75062	14351	14351	14351	13356	5702	55
W 16 °C AF R1	81171	68058	32326	32326	32326	32079	5702	228
W 16 °C AF R2	174353	161240	76121	76121	76121	75648	5702	179
W 16 °C AF R3	-	-	-	-	-	-	-	-
W 24 °C AF R1	51868	38755	18248	18248	18248	18080	5702	245
W 24 °C AF R2	55335	42222	19283	19283	19283	19120	5702	227
W 24 °C AF R3	39451	26338	12320	12320	12320	12183	5702	208
