# Supplementary material for: Implications of Climate Change: How Does Increased Water Temperature Influence Biofilm and Water Quality of Chlorinated Drinking Water Distribution Systems?
Source: Front Microbiol. 2021 Jun 8;12:658927. doi: 10.3389/fmicb.2021.658927 (PMC8217620; doi:10.3389/fmicb.2021.658927)
Supplement: Supplementary Table 4 — Water physico-chemical parameters measured on sampling days during the growth phase and after mobilisation phase (AM). All values represent an average of three water replicates analysis ± standard deviation. [file Table_4.PDF]

	16 °C				
**Sampling day**	**0**	**10**	**20**	**30**	**AM**
**Temperature (°C)**	15.7 ± 0.06	15.5 ± 0.07	15.9 ± 0.09	15.8 ± 0.15	15.6 ± 0.03
**pH**	7.59 ± 0.08	7.09 ± 0.11	6.78 ± 0.29	6.61 ± 0.16	6.53 ± 0.08
**Total Cl (mg/L)**	0.93 ± 0.06	0.19 ± 0.01	0.19 ± 0.01	0.22 ± 0.04	0.18 ± 0.02
**Free Cl (mg/L)**	0.89 ± 0.05	0.08 ± 0.01	0.11 ± 0.00	0.14 ± 0.02	0.18 ± 0.02
**Fe (µg/L)**	42.4 ± 0.65	41.3 ± 1.09	46.3 ± 0.92	44.6 ± 0.68	53.7 ± 2.09
**Mn (µg/L)**	0.33 ± 0.07	0.39 ± 0.02	0.52 ± 0.02	0.45 ± 0.00	0.69 ± 0.06
**TOC (mg/L)**	1.21 ± 0.00	1.21 ± 0.05	1.23 ± 0.05	1.13 ± 0.03	1.49 ± 0.04
	**24 °C**				
**Sampling day**	**0**	**10**	**20**	**30**	**AM**
**Temperature (°C)**	23.1± 0.41	24.0 ± 0.03	23.8 ± 0.07	23.9 ± 0.06	23.4 ± 0.03
**pH**	6.79 ± 0.19	7.47 ± 0.21	6.92 ± 0.09	6.80 ± 0.16	6.93 ± 0.13
**Total Cl (mg/L)**	0.80 ± 0.01	0.13 ± 0.03	0.13 ± 0.01	0.13 ± 0.05	0.21 ± 0.01
**Free Cl (mg/L)**	0.68 ± 0.03	0.08 ± 0.04	0.08 ± 0.01	0.10 ± 0.02	0.11 ± 0.03
**Fe (µg/L)**	49.0 ± 0.63	49.9 ± 0.54	59.3 ± 0.83	45.1 ± 1.07	81.2 ± 1.34
**Mn (µg/L)**	0.39 ± 0.01	0.47 ± 0.01	0.42 ± 0.02	0.39 ± 0.02	1.20 ± 0.04
**TOC (mg/L)**	1.35 ± 0.05	1.44 ± 0.06	1.34 ± 0.06	1.31 ± 0.02	1.50 ± 0.02
