# Supplementary material for: Implications of Climate Change: How Does Increased Water Temperature Influence Biofilm and Water Quality of Chlorinated Drinking Water Distribution Systems?
Source: Front Microbiol. 2021 Jun 8;12:658927. doi: 10.3389/fmicb.2021.658927 (PMC8217620; doi:10.3389/fmicb.2021.658927)
Supplement: Supplementary Table 5 — Results from the Mann-Whitney U test to determine statistical differences in bacterial and fungal alpha diversity indices between temperatures. Differences were considered statistically significant when p-value was ≤0.05. [file Table_5.PDF]

Mann-Whitney *U* test		Bacteria		
		**Chao1**	**Simpson**	**Shannon**
Biofilm	Day 10	W = 2<br>p-value = 0.4	W = 2<br>p-value = 0.4	W = 9<br>p-value = 0.01
	Day 20	W = 6<br>p-value = 0.35	W = 5<br>p-value = 0.5	W = 6<br>p-value = 0.35
	Day 30	W = 8<br>p-value = 0.1	W = 3<br>p-value = 0.35	W = 8<br>p-value = 0.1
	AF	W = 9<br>p-value = 0.05	W = 0<br>p-value = 0.06	W = 9<br>p-value = 0.05
Water	Day 0	W = 7<br>p-value = 0.9	W = 3<br>p-value = 0.8	W = 6<br>p-value = 0.7
	Day 10	W = 3<br>p-value = 0.8	W = 4<br>p-value = 0.5	W = 6<br>p-value = 0.35
	Day 20	W = 2<br>p-value = 0.2	W = 3<br>p-value = 0.35	W = 5<br>p-value = 0.5
	Day 30	W = 2<br>p-value = 0.2	W = 8<br>p-value = 0.1	W = 2<br>p-value = 0.2
	AF	W = 8<br>p-value = 0.1	W = 2<br>p-value = 0.2	W = 9<br>p-value = 0.05

Mann-Whitney *U* test		Fungi		
		**Chao1**	**Simpson**	**Shannon**
Biofilm	Day 10	W = 6<br>p-value = 0.35	W = 0<br>p-value = 0.05	W = 9<br>p-value = 0.05
	Day 20	W = 9<br>p-value = 0.05	W = 0<br>p-value = 0.05	W = 9<br>p-value = 0.05
	Day 30	W = 9<br>p-value = 0.05	W = 0<br>p-value = 0.02	W = 9<br>p-value = 0.35
	AF	W = 9<br>p-value = 0.05	W = 0<br>p-value = 0.05	W = 9<br>p-value = 0.05
Water	Day 0	W = 7<br>p-value = 0.06	W = 3<br>p-value = 0.95	W = 0<br>p-value = 0.1
	Day 10	W = 3<br>p-value = 0.5	W = 4<br>p-value = 0.35	W = 6<br>p-value = 0.35
	Day 20	W = 2<br>p-value = 0.5	W = 3<br>p-value = 0.35	W = 5<br>p-value = 0.5
	Day 30	-	-	-
	AF	W = 0<br>p-value = 0.05	W = 4<br>p-value = 0.5	W = 2<br>p-value = 0.2
